# Active Tuberculosis Is Characterized by Highly Differentiated Effector Memory Th1 Cells

**DOI:** 10.3389/fimmu.2018.02127

**Published:** 2018-09-19

**Authors:** Riccardo Arrigucci, Karim Lakehal, Pooja Vir, Deborah Handler, Amy L. Davidow, Rosa Herrera, Julia Dolores Estrada-Guzmán, Yuri Bushkin, Sanjay Tyagi, Alfred A. Lardizabal, Maria Laura Gennaro

**Affiliations:** ^1^Public Health Research Institute, New Jersey Medical School, Rutgers, The State University of New Jersey, Newark, NJ, United States; ^2^Global Tuberculosis Institute, New Jersey Medical School, Rutgers, The State University of New Jersey, Newark, NJ, United States; ^3^Department of Biostatistics, School of Public Health, New Jersey Medical School, Rutgers, The State University of New Jersey, Newark, NJ, United States; ^4^Facultad de Medicina Mexicali, Universidad Autónoma de Baja California, Mexicali, Mexico

**Keywords:** FISH-Flow, T cell activation, cytokine, immunophenotyping, single-cell gene expression, memory T cells, flow cytometry

## Abstract

Despite advances in diagnosing latent *Mycobacterium tuberculosis* infection (LTBI), we still lack a diagnostic test that differentiates LTBI from active tuberculosis (TB) or predicts the risk of progression to active disease. One reason for the absence of such a test may be the failure of current assays to capture the dynamic complexities of the immune responses associated with various stages of TB, since these assays measure only a single parameter (release of IFN-γ) and rely on prolonged (overnight) T cell stimulation. We describe a novel, semi-automated RNA flow cytometry assay to determine whether immunological differences can be identified between LTBI and active TB. We analyzed antigen-induced expression of Th1 cytokine mRNA after short (2- and 6-h) stimulation with antigen, in the context of memory T cell immunophenotyping. IFNG and TNFA mRNA induction was detectable in CD4+ T cells after only 2 h of *ex vivo* stimulation. Moreover, IFNG- and TNFA-expressing CD4+ T cells (Th1 cells) were more frequent in active TB than in LTBI, a difference that is undetectable with conventional, protein-based cytokine assays. We also found that active TB was associated with higher ratios of effector memory to central memory Th1 cells than LTBI. This effector memory phenotype of active TB was associated with increased T cell differentiation, as defined by loss of the CD27 marker, but not with T cell exhaustion, as determined by PD-1 abundance. These results indicate that single-cell-based, mRNA measurements may help identify time-dependent, quantitative differences in T cell functional status between latent infection and active tuberculosis.

## Introduction

Human infection with the intracellular pathogen, *Mycobacterium tuberculosis*, manifests itself in two forms. One is active TB, which most typically presents as a pulmonary disease that is contagious (1, 2). The other is latent infection (LTBI), which is characterized by immune sensitization to *M. tuberculosis* antigens in the absence of clinical symptoms (3). Diagnostic methods exist to identify active TB and LTBI. These are based on detection of mycobacteria and/or mycobacterial components as a sign of active TB (4) and of antigen-specific T cell responses to *M. tuberculosis* antigen stimulation *in vivo* or *ex vivo* for LTBI (5). Unfortunately, even the most accurate *ex vivo* LTBI assays, which measure IFN-γ release by antigen-stimulated peripheral T cells (Interferon gamma release assays-IGRA), do not distinguish between LTBI and active TB, nor do they provide information on the risk of reactivation and progression to disease (6–8). Attaining such a distinction would greatly impact TB control, because it would help identify high-risk subjects for LTBI therapy in low-resource settings and consequently reduce the risk of disease reactivation and transmission of infection. New tools distinguishing LTBI from active TB based on host responses are sorely needed.

The multifactorial nature of the progression from chronic asymptomatic infection to active disease likely underlies the inadequacy of single-parameter assays, such as the IGRAs, as predictive tools of TB reactivation (9). Multi-parameter, T-cell-based assays have addressed either production of multiple cytokines (10–12) or memory phenotypes and expression of activation markers (13–22). Some of these studies have generated potentially promising results [for example, (22)], supporting the possibility that host signatures of infection stage or immunological protection can be identified.

A daunting challenge is that the demarcation between latent and active TB is blurred. Given the chronic nature of *M. tuberculosis* infection, asymptomatic and symptomatic infection stages map along a continuum of host and pathogen responses that ultimately determine outcome (8). Thus, it is conceivable that an accurate definition of specific states along this continuum requires combined analysis of qualitative, quantitative, and temporal aspects of the host response. New analytical methodologies may be needed to dissect the temporal complexity of the T cell response to *M. tuberculosis* infection.

One possible approach for studying the time scale of the T cell response is to use mRNA as readout, since mRNA is typically more rapidly induced than protein in response to stimulus and has a shorter half-life than the corresponding protein. In a previous proof-of-principle study we demonstrated that RNA flow cytometry, which allows for multi-parameter, concurrent analysis of mRNA and protein in the same cell (23–25), is applicable to the detection of antigen-specific T cell responses to *M. tuberculosis* antigens (26). Here, we utilized a semi-automated RNA flow cytometry platform (24) to determine whether a multi-parametric (mRNA and protein) assay for T cell memory phenotypes and cytokine production identifies differences between LTBI and active TB.

## Materials and methods

### Study population and enrollment

Study participants between 19 and 72 years of age having active TB were enrolled during the period of September 2014–January 2017 from two county clinics in New Jersey, USA (*n* = 19) and at the Autonomous University of Baja California (UABC) in Mexicali, Baja California, Mexico (*n* = 15) after written consent was obtained. Participants became eligible when investigated for active TB. Final diagnosis of pulmonary TB was based on microbiological results (presence of acid-fast bacilli in sputum and sputum culture positivity). Active TB patients were recruited within 56 days of initiation of antibiotic therapy. Diagnosis of LTBI was based on the results of QuantiFERON-TB Gold assay (Qiagen), performed according to manufacturer's instructions. All LTBI+ subjects had a negative chest x-ray. All study subjects were HIV negative. Demographic characteristics of the study population are shown in Supplementary Table 1.

### PBMC stimulation

Peripheral blood mononuclear cells (PBMC) were isolated by Ficoll density gradient and stored in liquid nitrogen until processed, as previously described (26). Cryopreserved PBMC were thawed and washed in pre-warmed RPMI 1640 supplemented with 2 mM L-glutamine, 10% FBS, 100 U/ml penicillin, and 100 μg/ml streptomycin (all from Corning cellgro, Manassas, VA) (complete RPMI). PBMC were resuspended in complete RPMI containing 0.1 μg/ml CD28/CD49d antibodies (BD Biosciences, San Jose, CA, United States) at a cell concentration of 2 to 3 × 10^6^/ml. After the addition of CD28/CD49d antibodies, 1-ml aliquots of the cell suspension were transferred in 12 × 75 mm polystyrene FACS tubes and either stimulated with 10 μg/ml PPD (Staten Serum Institute, Copenhagen, Denmark) or left unstimulated. A mixture of 25 ng/ml phorbol 12-myristate 13-acetate (PMA) (Sigma-Aldrich, St. Louis, MO, United States) and 0.5 μM ionomycin calcium salt (Enzo Life Sciences, Farmingdale, NY, United States) served as positive control for T cell stimulation. Tubes were incubated for 2 or 6 h at 37°C in a 5% CO_2_ humidified atmosphere.

### Semi-automated fish-flow assay

Cytokine mRNA was measured utilizing a semi-automated RNA flow cytometry assay (FISH-Flow), as previously described (24). After stimulation, samples were processed in a modified version of the Lyse Wash Assistant (LWA) instrument (BD Biosciences, San Jose, CA, United States), which reduces processing time, cell loss, and variability among samples and operators (24). Briefly, tubes were transferred in the LWA carousel, washed, incubated for 5 min with 5 μl FcX (Fc receptor solution, BioLegend, San Diego, CA, United States) and stained with a mix of the following fluorochrome-conjugated antibodies: CD3 FITC (Clone HIT3a, BD Biosciences), CD4 BUV496 (Clone SK3, BD Biosciences), CD8 BUV395 (Clone RPA-T8, BD Biosciences), CD45RA BV421 (Clone HI100, BD Biosciences), CCR7 PE (Clone G043H7, Biolegend), CCR6 PE-Cy7 (Clone G034E3, Biolegend), CXCR3 PE-Dazzle594 (Clone G025H7, Biolegend), CD27 BUV395 (Clone L128, BD Biosciences), PD-1 PE-Dazzle594 (CD279, Clone EH12.2H7, Biolegend) CD69 Alexa700 (Clone FN50, BD Biosciences). After staining, cells were washed in PBS-FISH buffer (0.2 mg/ml BSA in 1 × PBS), fixed in 4% (vol/vol) paraformaldehyde in 1 × PBS for 30 min at room temperature (RT), and washed again in PBS-FISH. Cells were permeabilized in 0.2% (vol/vol) Tween 20 in 1 × PBS for 30 min at RT, washed in hybridization wash buffer (HWB, 2 × saline sodium citrate, 10% (vol/vol) formamide, 0.2 mg/ml BSA), and hybridized overnight in a final volume of 400 μl of hybridization buffer [10% (wt/vol) dextran sulfate, 2 × saline sodium citrate, 10% (vol/vol) formamide, 0.2 mg/ml BSA, 1 mg/ml tRNA] containing 10 ng of Cy5 conjugated sm-FISH probes for the target cytokine mRNA. After hybridization, cells were washed in HWB and analyzed by flow cytometry.

### Flow cytometry

Data were acquired in a BD Fortessa X-20 flow cytometer (BD Biosciences, San Jose, CA) and analyzed with FlowJo 10.2 software (FlowJo). BD™ Cytometer Setup & Tracking beads (CS&T, BD Biosciences) were used to calibrate the flow cytometer before each experiment. A total of 250,000–500,000 events were acquired per sample. Polystyrene compensation beads (BD Biosciences) were used to calculate spectral overlap values for each fluorochrome, according to the manufacturer's instructions. The following lasers, band pass (BP) and long pass (LP) filters were used for each fluorochrome: BUV395 and BUV496, 355 nm laser and 379/28 BP and 410 LP, 513/30 BP, respectively; BV421, 405-nm laser and 450/50 BP; FITC, 488-nm laser and 505 LP, 530/30 BP; PE, PE-CF594, PE-Cy7, 561-nm laser and 586/15 BP, 595 LP and 610/20 BP, 750 LP and 780/60 BP, respectively; Cy5, 640-nm laser, and 670/30 BP.

### ELISA

PBMC were stimulated with 10 μg PPD in 24-well tissue culture plates for 6 h. Supernatants were collected by centrifugation and stored frozen at −80°C. Levels of IFN-γ in culture supernatants were assayed using commercial ELISA kits [OptEIA™ Human IFN-γ ELISA kit II (BD Biosciences, San Jose, CA, United States)], according to manufacturer's instructions.

### Quantiferon TB-gold assay

The QuantiFERON TB-Gold assay (Qiagen) was performed according to the instructions provided by the manufacturer. Plasma collected from each QuantiFERON tube was stored at −80°C prior to analysis.

### Statistical analysis

Data were analyzed with SAS version 9.3. Since the data were not normally distributed, two non-parametric tests were used: the Wilcoxon two-sample test was used to analyze the inter-group differences between LTBI and active TB donors, while the Wilcoxon signed rank test was used to analyse intra-group differences between time points. Significance was set as *P* < 0.05. No data were excluded from the analysis. The data were visualized using GraphPad Prism 7. The box plots were drawn to show the 25th percentile, the median, and the 75th percentile of the distribution. The upper and lower whiskers represent the 90th and 10th percentile. Extreme values (below the 10th percentile and above the 90th percentile) are shown as (•) symbol.

## Results

### The number of activated CD4+ T cells inducing Th1 cytokine genes in response to antigen stimulation is higher during active TB than in LTBI

In a previous study, we showed that production of IFNG and TNFA mRNA, in response to *ex vivo* antigen stimulation associated with LTBI, was mostly found in CD4+ T cells (>90%), with negligible contribution by other lymphocyte subsets (26). Thus, we investigated CD4+ T cell production of two Th1 cytokine mRNAs, IFNG and TNFA, following stimulation of peripheral blood mononuclear cells (PBMC) from donors diagnosed with either active TB or LTBI. As *M. tuberculosis* antigen we used purified protein derivative (PPD), a complex, multi-epitope protein mixture (27), to capture a broad T cell response repertoire, which may be more representative of infection-state specific responses than the response to a small number of epitopes (28, 29). To capture temporal aspects of the response, antigen stimulation was conducted for 2 and 6 h.

**Figure 1:**
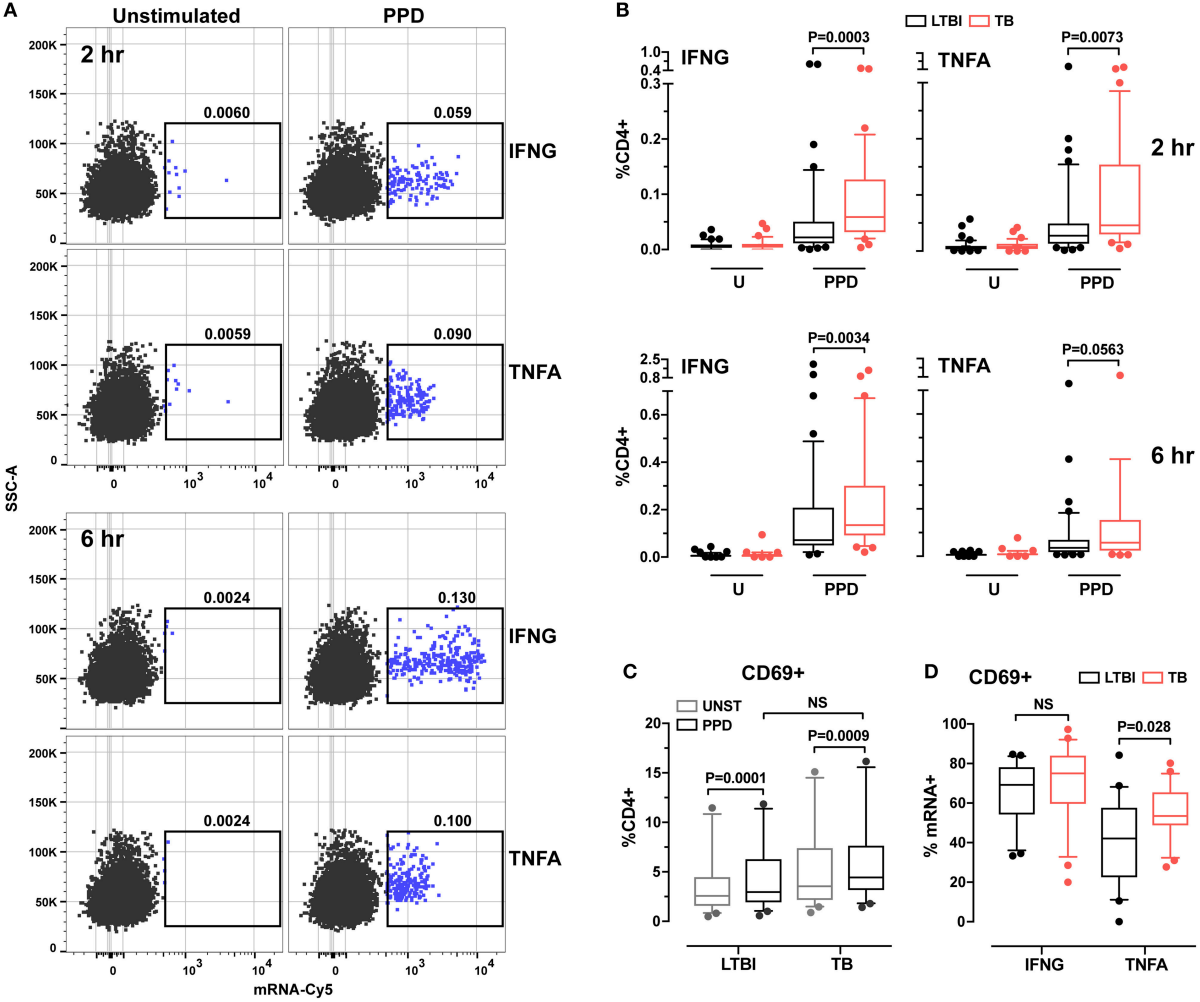
Analysis of cytokine expression and cell activation in CD4+ T cells from LTBI and active TB donors. **(A)** Representative dot plots of IFNG and TNFA cytokine expression in CD4+ T cells after 2 and 6 h *in vitro* stimulation. PBMC were stimulated for 2 and 6 h with PPD or left unstimulated. After stimulation samples were stained for surface markers and hybridized with IFNG or TNFA mRNA probes. Frequencies of CD4+ cytokine mRNA+ cells are reported above each gate. **(B)** Cytokine expression in CD4+ cells. Data are expressed as the frequencies of CD4+ T cells expressing IFNG or TNFA mRNA at each time point. LTBI *n* = 47, TB *n* = 34. **(C)** CD69 expression in unstimulated and PPD stimulated CD4+ T cells for 2 h. Data were expressed as frequency of CD4+ T cells. LTBI *n* = 24, TB *n* = 22. Gating strategy is described in Supplementary Figure 1C (upper panel). Outliers above 20% (one per group) were removed to allow for better data representation. **(D)** CD69 expression in IFNG and TNFA mRNA+ CD4+ T cells. Data were expressed as frequency of total IFNG+ or TNFA+ cells after 2 h of PPD stimulation. LTBI *n* = 24, TB *n* = 22. Gating strategy is described in Supplementary Figure 1C (lower panel). The Wilcoxon two-sample test was used to analyze differences between LTBI and active TB donors. The box plots show lower quartile, median, and upper quartile of the distribution. The upper and lower whiskers represent the 90th and 10th percentile. Extreme values (below the 10th percentile and above the 90th percentile) are shown as (•) symbol.

When we analyzed the data obtained from LTBI (*n* = 47) and active TB donors (*n* = 34), we found that antigen-induced production was detectable at 2 h post-stimulation for both cytokine mRNAs (a representative dot plot is shown in Figure 1A; gating strategies are found in Supplementary Figure 1). The temporal profiles of induction differed between the two cytokine genes: in both diagnostic groups, IFNG mRNA levels—but not those of TNFA mRNA—continued to increase between 2- and 6-h post-stimulation (Supplementary Figure 2); mRNA production reflected production of the corresponding protein (see IFNG example in Supplementary Figure 3), as expected (30). When we calculated the frequencies of antigen-specific CD4+ T cells expressing IFNG or TNFA, we found they were higher in active TB than in LTBI donors (*P* = 0.0003 and *P* = 0.0073, respectively, per cytokine gene) (Figure 1B). After 6 h of stimulation, frequencies were still higher for IFNG mRNA (*P* = 0.0034) in active TB than in LTBI, while the TNFA comparison lost significance (*P* = 0.056; Figure 1B). Thus, the RNA-based assay revealed differences between the two groups. In contrast, the results of conventional assays, in which cytokine release is measured after overnight stimulation, were indistinguishable between the two groups (Supplementary Figure 4), presumably due to longer protein half-life and protein accumulation. These data strongly imply that RNA flow cytometry provides a more dynamic representation of the Th1 response to stimulus than the standard assay.

Since cytokine responses were detectable at 2 h post-stimulation, we investigated the relationship between T cell activation state and cytokine production by examining the expression of CD69, a cell surface marker that becomes detectable shortly after T cell receptor engagement (31). We found that, after 2 h of PPD stimulation, CD69 expression in CD4+ T cells increased in both donor groups, with no difference between groups (Figure 1C). When we analyzed the frequencies of CD4+CD69+ cells among cytokine mRNA-expressing cells, we found that CD69 expression differed between TNFA+ cells from the two donor groups (*P* = 0.028), but not among IFNG+ cells (*P* > 0.05; Figure 1D). No difference was observed for either cytokine at 6 h post-stimulation between the two groups (data not shown). These results suggest that the TNFA-expressing T cells differ functionally between active TB and LTBI.

### Th1 cells are characterized by a dominant effector memory phenotype during active Tb

**Figure 2:**
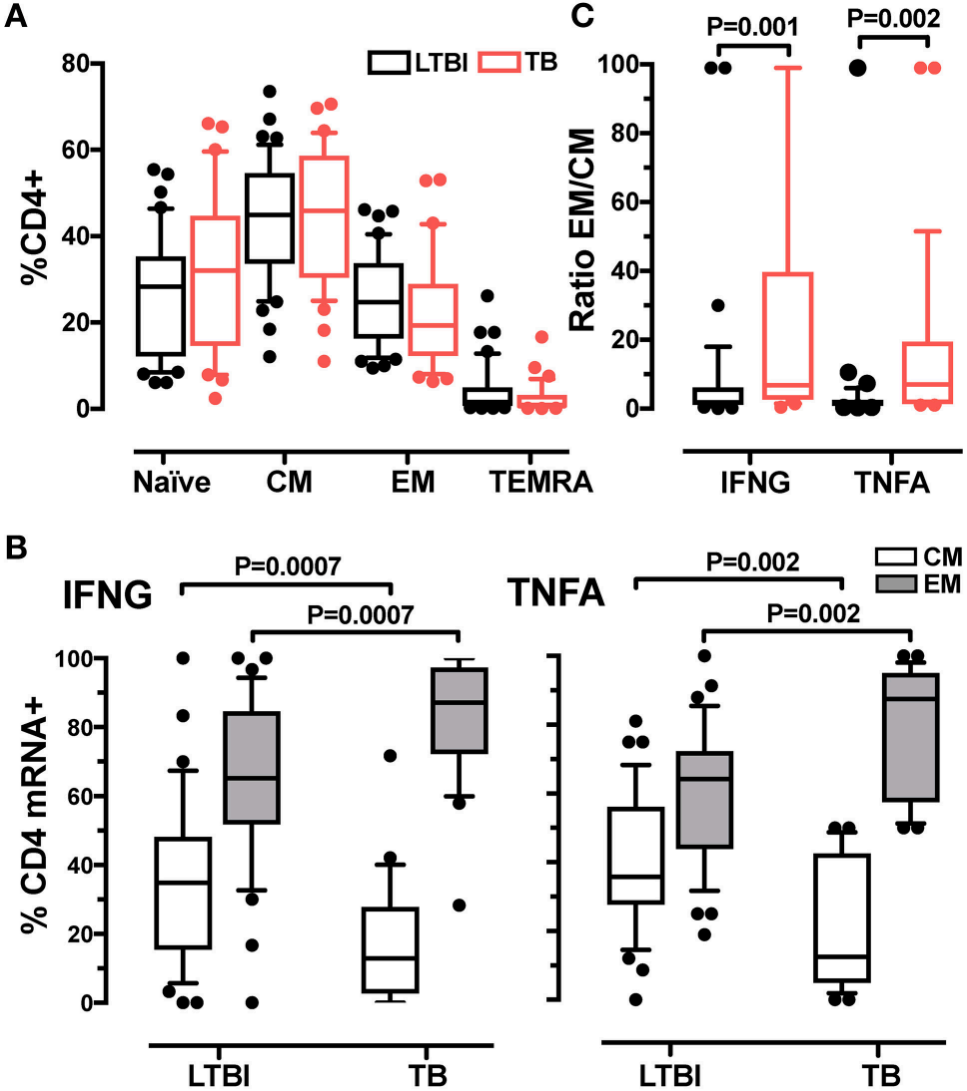
Analysis of memory CD4+ T cell subsets in LTBI and active TB donors. **(A)** Frequencies of CD4+ T cell subsets in LTBI and active TB patients. Subsets were defined according to surface expression of CD45RA and CCR7 markers. Data were expressed as the mean of the frequency of CD4+ T cells in unstimulated and stimulated samples at 2 and 6 h. LTBI *n* = 39, TB *n* = 31. **(B)** Cytokine expression in effector and central memory T cells from LTBI and active TB donors. Data were expressed as frequency of the total memory T cells expressing IFNG or TNFA after 2 h of PPD stimulation. LTBI *n* = 37, TB *n* = 29. **(C)** EM/CM ratio. Data are expressed as the ratio of the frequencies of EM and CM T cells expressing IFNG or TNFA mRNA from LTBI and active TB patients after 2 h of PPD stimulation. LTBI *n* = 37, TB *n* = 29. Similar results were observed at the 6-h time point (data not shown). Gating strategy utilized for memory markers analysis is described in Supplementary Figures 5A,B. The Wilcoxon two-sample test was used to analyse differences between LTBI and active TB donors. The box plots show lower quartile, median, and upper quartile of the distribution. The upper and lower whiskers represent the 90th and 10th percentile. Extreme values (below the 10th percentile and above the 90th percentile) are shown as (•) symbol.

The more rapid response detected in active TB than in LTBI suggested differences in memory phenotypes between CD4+ T cells in the two donor groups. We tested this possibility by characterizing T cell memory phenotypes. By using the CD45RA and CCR7 markers, we distinguished four memory CD4+ T cell subsets: naïve (CD45RA^+^CCR7^+^), central memory (CM, CD45RA^−^CCR7^+^), effector memory (EM, CD45RA^−^CCR7^−^) and terminally differentiated effector memory (TEMRA, CD45RA^+^CCR7^−^) (32, 33) (Supplementary Figure 5A). We first determined that the relative frequencies of the four subsets in the total CD4+ T cell population did not differ between LTBI and active TB groups (Figure 2A). When we analyzed CD4+ T cells positive for cytokine mRNA, we found that the majority (>85%) of IFNG+ or TNFA+ cells were contained in the CM and EM subsets (Supplementary Figure 6). Differences were observed between donor groups: for both cytokine mRNA-expressing cells, the frequencies of the CM subset were lower and those of the EM subset were higher in the active TB group than in the LTBI group (Figure 2B) (no differences between the two groups were found in the naïve and TEMRA subsets; not shown). The EM/CM ratio was, on average, 3-fold higher in active TB than in LTBI donors for IFNG and TNFA mRNA-positive cells (*P* = 0.001 and *P* = 0.002, respectively) (Figure 2C). These results indicate that the memory CD4+ T cell pool in active TB is skewed toward an effector memory phenotype.

### EM Th1 cells are more differentiated during active Tb than in LTBI

**Figure 3:**
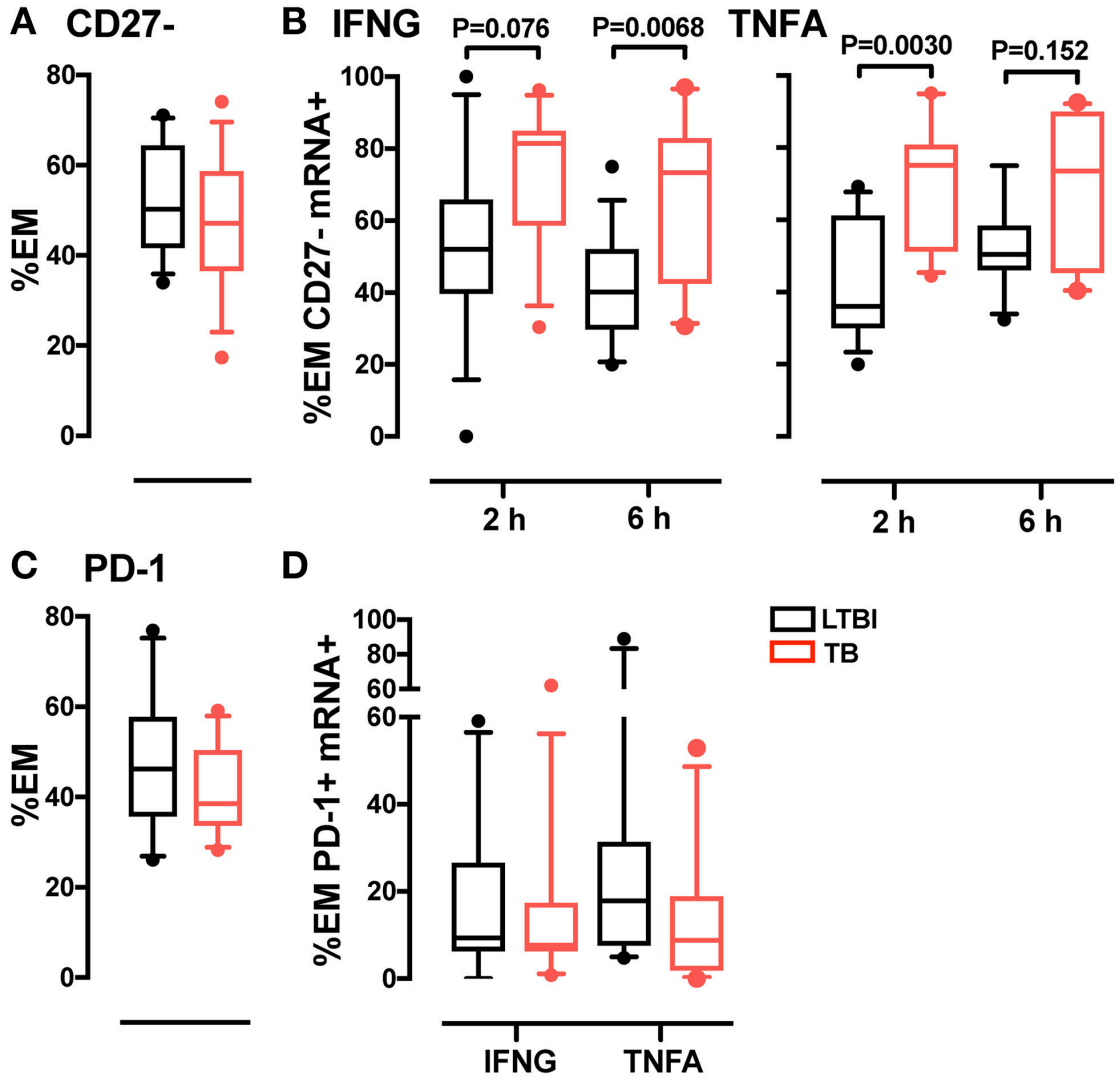
Analysis of CD27 and PD-1 expression in CD4 EM memory T cells. **(A)** CD27 expression in EM CD4 T cells. Data from each patient were plotted as the mean of the frequency of total EM CD4 T cells in unstimulated and stimulated samples at 2 and 6 h. LTBI *n* = 16, TB *n* = 15. **(B)** Frequency of CD27- memory T cells in IFNG and TNFA expressing cells. Data were expressed as frequency of the total IFNG or TNFA expressing cells in EM CD4 T cells from donors after 2 and 6 h of PPD stimulation. LTBI *n* = 16, TB *n* = 15. **(C)** PD-1 expression in EM CD4 T cells. Data were expressed as the mean of the frequency of total EM T cells in unstimulated and stimulated samples at 2 and 6 h. LTBI *n* = 10, TB *n* = 11. **(D)** PD-1 expression in EM CD4 T cells expressing IFNG and TNFA mRNA. Data were expressed as the frequency of the total IFNG or TNFA expressing cells in EM subsets at 6 h. Similar results were observed at the 2-h time point (data not shown). LTBI *n* = 10, TB *n* = 11. Gating strategy utilized for CD27 and PD-1 analysis is described in Supplementary Figures 5C,D. The Wilcoxon two-sample test was used to analyze differences between LTBI and active TB donors. The box plots show lower quartile, median, and upper quartile of the distribution. The upper and lower whiskers represent the 90th and 10th percentile. Extreme values (below the 10th percentile and above the 90th percentile) are shown as (•) symbol.

One marker of memory T cell differentiation is the downregulation of the co-stimulatory receptor CD27 (34, 35), which is associated with diminished proliferative capacity (36) and high antigen recall response (37). When we tested the CD4+ EM subset for expression of CD27, we observed similar frequencies of CD27+ cells (~50%) in the active TB and LTBI donor groups (Figure 3A). However, among the cytokine mRNA-expressing cells, the frequencies of CD27- cells were higher in active TB than in LTBI donors (Figure 3B). These differences were statistically significant at 6-hrs post-stimulation for IFNG+ cells (*P* = 0.0068) and at 2-hrs for TNFA+ cells (*P* = 0.003; Figure 3B), in concert with the peak expression of the corresponding cytokine mRNA (Supplementary Figure 2). Thus, the Th1 cell response during active TB is characterized by a highly differentiated effector memory pool.

### PD-1 expression in EM Th1 cells does not differ between active and latent infection

A marker associated with antigen-mediated T cell activation is programmed cell death 1 (PD-1), a surface protein that regulates T cell responses during cancer and chronic infection, where persistent antigen stimulation may lead to exhaustion (38). To determine whether T cell differentiation was associated with exhaustion in tuberculosis, we analyzed PD-1 expression in CD4+ memory T cells from active TB and LTBI donors. We found that the frequency of PD-1+ cells in the EM subset did not vary between the two donor groups, regardless of cytokine expression (Figures 3C,D). Thus, active TB is not associated with greater memory T cell exhaustion than latent infection.

### Most of the Th1 cell subset during LTBI and active Tb is CXCR3^+^CCR6^+^

**Figure 4:**
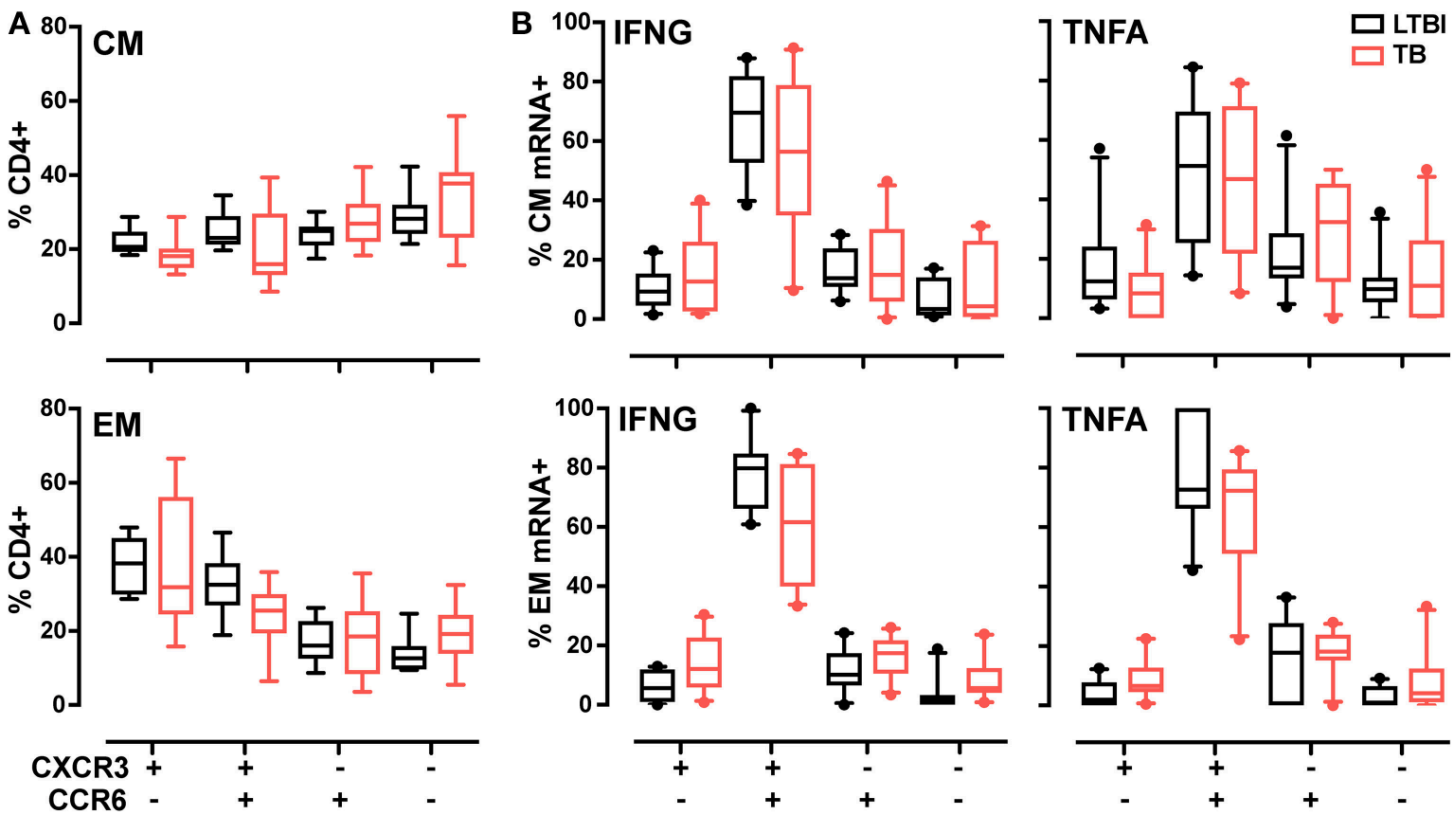
CXCR3, CCR6 T memory subsets in LTBI and active TB donors. **(A)** Frequencies of CXCR3 and CCR6 subsets in CM and EM CD4+ T cells. Data from each patient were plotted as the mean of the frequency of total CM or EM CD4+ cells in unstimulated and stimulated samples at 2 and 6 h. **(B)** Cytokine expression in CXCR3 and CCR6 subsets in CM and EM CD4+ T cells. Data are expressed as the frequency of total IFNG or TNFA expressing cells after 6 h of PPD stimulation. Similar results were observed at the 2-h time point (data not shown). LTBI *n* = 10, TB *n* = 10. Gating strategy is reported in Supplementary Figures 7A,B. The Wilcoxon two-sample test was used to analyze differences between LTBI and active TB donors. The box plots show lower quartile, median, and upper quartile of the distribution. The upper and lower whiskers represent the 90th and 10th percentile. Extreme values (below the 10th percentile and above the 90th percentile) are shown as (•) symbol.

Another class of functional markers is constituted by chemokine receptors, which allow cells to extravasate and migrate to the sites of infection and inflammation. In T cells, the chemokine receptors vary with T helper function. For example, Th1 cells are characterized by surface expression of CCR5 and CXCR3 (39), while CCR6 and CCR4 characterize Th17 cell subset (40). Previous work has shown that, during LTBI, antigen-specific CD4+ T cells express the unique chemokine signature CCR6^+^CXCR3^+^ (40, 41); moreover, this signature is found both in Th1 and Th17 cells (42). Since it was unknown whether the frequency of this signature varies with TB diagnostic state, we compared expression of CXCR3 and CCR6 in the memory T cell pool in active TB and LTBI. Analysis of the four subsets identified by expression of these two markers (see Supplementary Figures 7A,B for the gating strategy) showed similar frequencies for all subsets in the total CD4+ CM and EM population in the two donor groups (Figure 4A). When cytokine mRNA-expressing cells were analyzed, the CCR6^+^CXCR3^+^ subset was most represented (~50–80%, on average) in both memory pools and for both cytokines (Figure 4B). No difference was observed between donor groups (Figure 4B). Thus, the Th1 cell pool does not change in terms of CXCR3 and CCR6 expression during the various stages of *M. tuberculosis* infection.

## Discussion

The present study showed that RNA flow cytometry affords rapid detection of cytokine mRNA induction in *ex vivo* stimulated CD4+ T cells. Such rapid detection makes it possible to identify differences between active tuberculosis and latent infection: more CD4+ T cells produced IFNG or TNFA mRNA as early as 2 h post-stimulation in active TB. This difference likely reflects the higher proportion of cytokine-producing cells with a highly differentiated EM phenotype (CD45RA^−^CCR7^−^CD27^−^) in active TB, since EM cells can mount faster and stronger responses to antigen recall than CM cells (43). Greater differentiation does not appear to result in exhaustion of Th1 cells, since PD1 expression did not differ between the two infection states. Our results also revealed differences between IFNG and TNFA induction profiles and greater proportions of CD4+CD69+ T cells producing TNFA (but not IFNG) during active TB relative to latent infection. These results imply functional differences among TNFA-producing cells in the two stages of *M. tuberculosis* infection. No difference between the two diagnostic groups was detected by conventional cytokine protein detection after overnight stimulation with antigen, in agreement with conclusions reached by a vast body of work [reviewed in (44, 45)]. Thus, our results strongly point to rapid detection of T cell responses for classification of *M. tuberculosis* infection states; they also show the appropriateness of RNA flow cytometry to detect these responses.

Our work provides new insight into dynamic aspects of the Th1 cell responses to *M. tuberculosis* infection. For example, previous work showed that, among Th1 cells, the proportion of CM cells exceeded that of EM cells during latent infection, while the situation was reversed in active disease (18, 46, 47). In sharp contrast, we report that the proportion of EM Th1 cells is higher than that of CM Th1 cells in both infection states. This result likely derives from our ability to detect early induction of cytokine mRNA in recall assays. We also found that the proportions of EM Th1 cells are higher and those of CM Th1 cells are lower in active TB than during latent infection, which likely explains why the rapid cytokine response of CD4+ T cells is higher in active TB than in latent infection. Consequently, the most informative parameter of the Th1 cell response is the ratio EM/CM rather than the predominant representation of a particular memory phenotype, at least among those tested in this study.

A dynamic view of the immune response may help explain inconsistencies of previous work. For example, some studies correlated polyfunctional T cells with active TB (16, 18, 20, 47–49) and others with latent infection (50, 51). Since human T cells release cytokines sequentially (52) and since the temporal production profiles vary with different cytokines [this work and (23, 26)], the levels of one or more cytokine per cell (as determined, e.g., by ELISPOT or intracellular staining) may vary with the time of sampling and reflect accumulation rather than production rate. Refinements of RNA flow cytometry are needed to directly test this possibility by concurrent analysis of multiple cytokine mRNAs.

In conclusion, our data show that detecting mRNA as analyte provides a more rapid and dynamic representation of the cytokine response of memory CD4+ T cells to antigen stimulation *ex vivo* than that afforded by conventional, protein-based detection methods. The mRNA responses, which are more vigorous in active TB than in latent infection, seem to be associated with highly differentiated—but not exhausted—EM cells. Our work also underscores that cellular signatures associated with clinical status may be time-dependent; failure to account for these dynamic properties may lead to incorrect interpretations. Our present results point to the ratio EM/CM among Th1 cells as a notable difference between active tuberculosis and latent infection. Although translating the quantitative differences we observed into diagnostic parameters and identifying the appropriate diagnostic platform will be challenging, our results constitute a clear first step toward the application of T cell memory phenotypes as elements of a host bio-signature of tuberculosis reactivation risk.

## Ethics statement

Adult (>18 years of age) subjects were screened and enrolled in this study after written informed consent was obtained at the Waymon C Lattimore Practice of the New Jersey Medical School in Newark, NJ, the Middlesex County Chest Clinic, or the Autonomy University of Baja California (UABC) in Mexicali, Baja California, Mexico. This study was conducted under a protocol approved by the Universidad Autónoma De Baja California College of Medicine and Psychology Bioethics Committee and the Rutgers University Health Sciences Institutional Review Board.

## Author contributions

RA, KL, PV, and MG: Conceived and designed experiments; RA, KL, and PV: Performed the experiments; RA and AD: Performed data analysis; DH, AL, RH, JE-G, and MG: Coordinated patient recruitment and obtained blood samples; YB, ST, and MG: Contributed the initial Fish-Flow protocol; MG: Supervised the study; RA and MG: Wrote the manuscript. All authors contributed to manuscript revision, read and approved the submitted version.

### Conflict of interest statement

Rutgers University receives royalties from the sale of prelabeled sm-FISH probes by Biosearch Technologies, which markets them as Stellaris probes. A fraction of these proceeds is distributed to ST's laboratory for research and to him personally. Additional patent applications related to this technology coauthored by YB, MG, and ST are pending. These proceeds, affiliations, and patent applications do not influence the conclusions of this research. The remaining authors declare that the research was conducted in the absence of any commercial or financial relationships that could be construed as a potential conflict of interest.
