# Supplementary material for: Active Tuberculosis Is Characterized by Highly Differentiated Effector Memory Th1 Cells
Source: Front Immunol. 2018 Sep 19;9:2127. doi: 10.3389/fimmu.2018.02127 (PMC6156157; doi:10.3389/fimmu.2018.02127)
Supplement: Supplementary file 1 [file Data_Sheet_1.pdf]

Supplementary Material

Active tuberculosis is characterized by highly differentiated effector memory Th1 cells

Riccardo Arrigucci, Karim Lakehal, Pooja Vir, Deborah Handler, Amy L. Davidow, Rosa Herrera, Julia Dolores Estrada-Guzmán, Yuri Bushkin, Sanjay Tyagi, Alfred A. Lardizabal, Maria Laura Gennaro*

***Correspondence**
Maria Laura Gennaro, MD
e-mail: marila.gennaro@rutgers.edu
phone: +1-973-854-3210

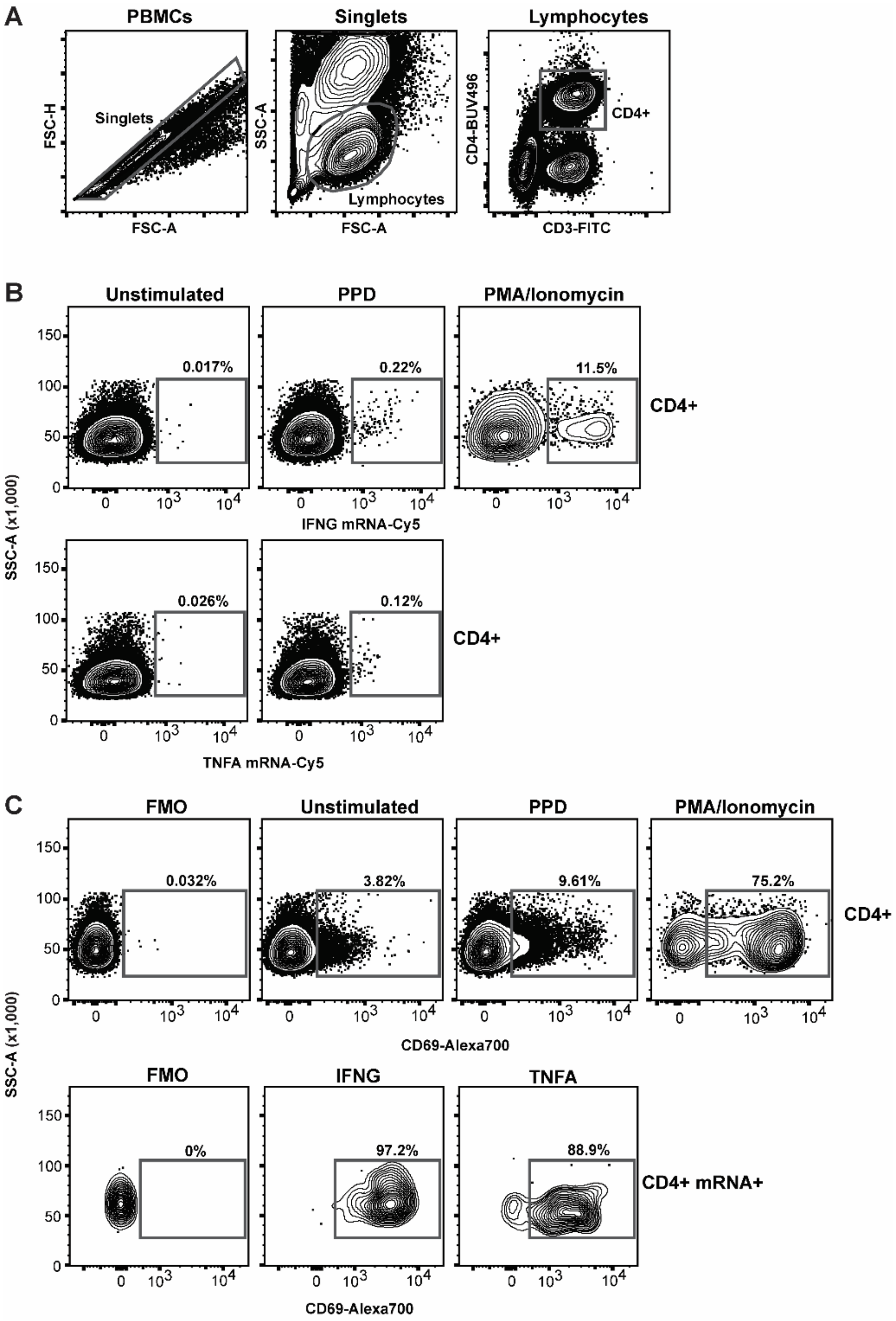

Supplementary Figure 1. Representative gating strategy utilized for the identification of cytokine expressing cells and analysis of CD69 surface expression. **(A)** Cell doublets were excluded from PBMC population using forward scatter area (FSC-A) and height (FSC-H); the lymphocyte population was gated according to FSC-A and side scatter area (SSC-A) parameters prior to the selection of CD4+ T cells. **(B)** Representative dot plots from an active TB donor showing IFNG and TNFA expression in CD4+ T cells after 6 hrs of PPD stimulation. **(C)** Representative dot plots showing CD69 expression in CD4+ T cells after 6 hrs of PPD and 2 hrs of PMA/Ionomycin (positive control) stimulation (upper panel) and CD69 expression in IFNG and TNFA expressing cells after 6 hrs of PPD stimulation (lower panel). Gates were defined using unstimulated negative controls and Fluorescence Minus One (FMO) controls.

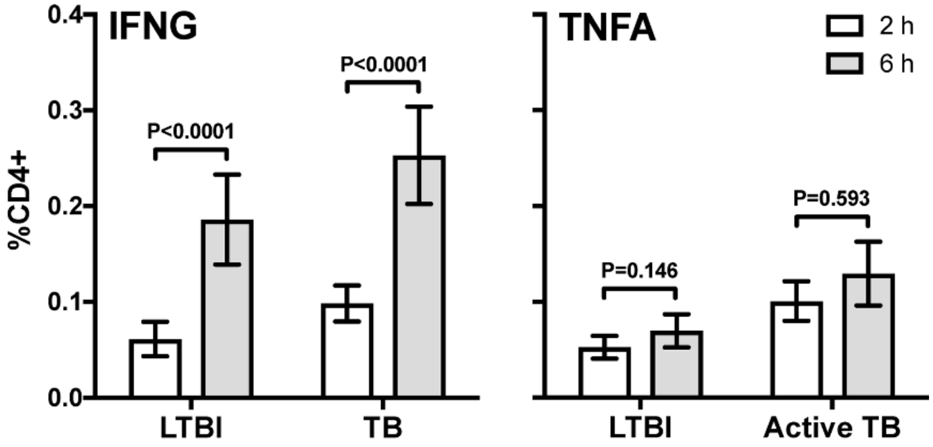

Supplementary Figure 2. IFNG and TNFA cytokine expression. Bars shows the frequencies of cytokine expressing CD4 T cells in LTBI and active TB donors after 2 and 6 hrs of PPD stimulation. LTBI n=47, TB n=34. Wilcoxon signed rank test was used to analyze intra-group differences between time points. Data represent the mean ± SEM.

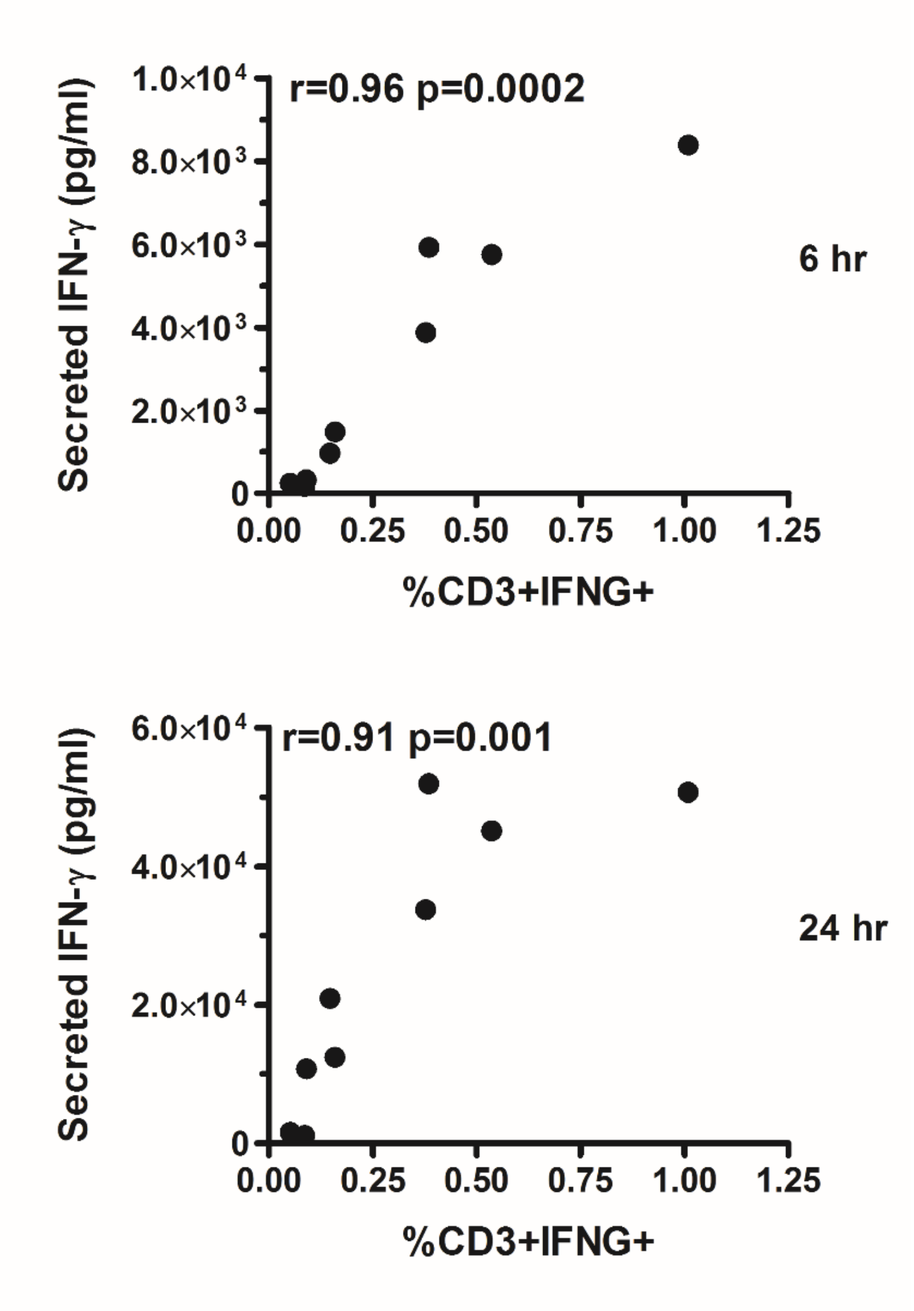

Supplementary Figure 3. Correlation of cytokine mRNA and protein production. PBMC from LTBI donors (n=9) were in-vitro stimulated with PPD for 6 and 24 hrs. IFNG expressing cells and levels of IFN-γ secreted protein were analyzed by FISH-Flow and ELISA assays, respectively. Data were analyzed by the Spearman's correlation test.

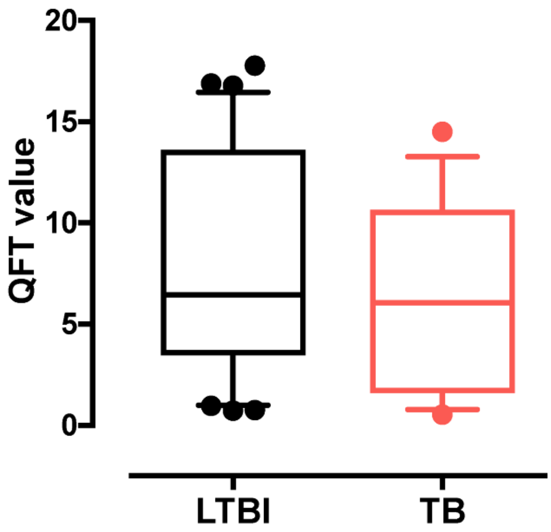

Supplementary Figure 4. QuantiFERON-TB Gold ELISA of LTBI and active TB patients. One ml of blood was incubated for 24 hrs in three QFT tubes (Nil, TB-antigen, Mitogen) per sample. After centrifugation, plasma was stored at -80˚C until analysis. QFT ELISA values were calculated as per instructions by the manufacturer. LTBI n=34, TB n=16. The Wilcoxon two-sample test was used to analyze differences between LTBI and active TB donors. The box plots show lower quartile, median, and upper quartile of the distribution. The upper and lower whiskers represent the 90th and 10th percentile. Extreme values (below the 10th percentile and above the 90th percentile) are shown as (•) symbol.

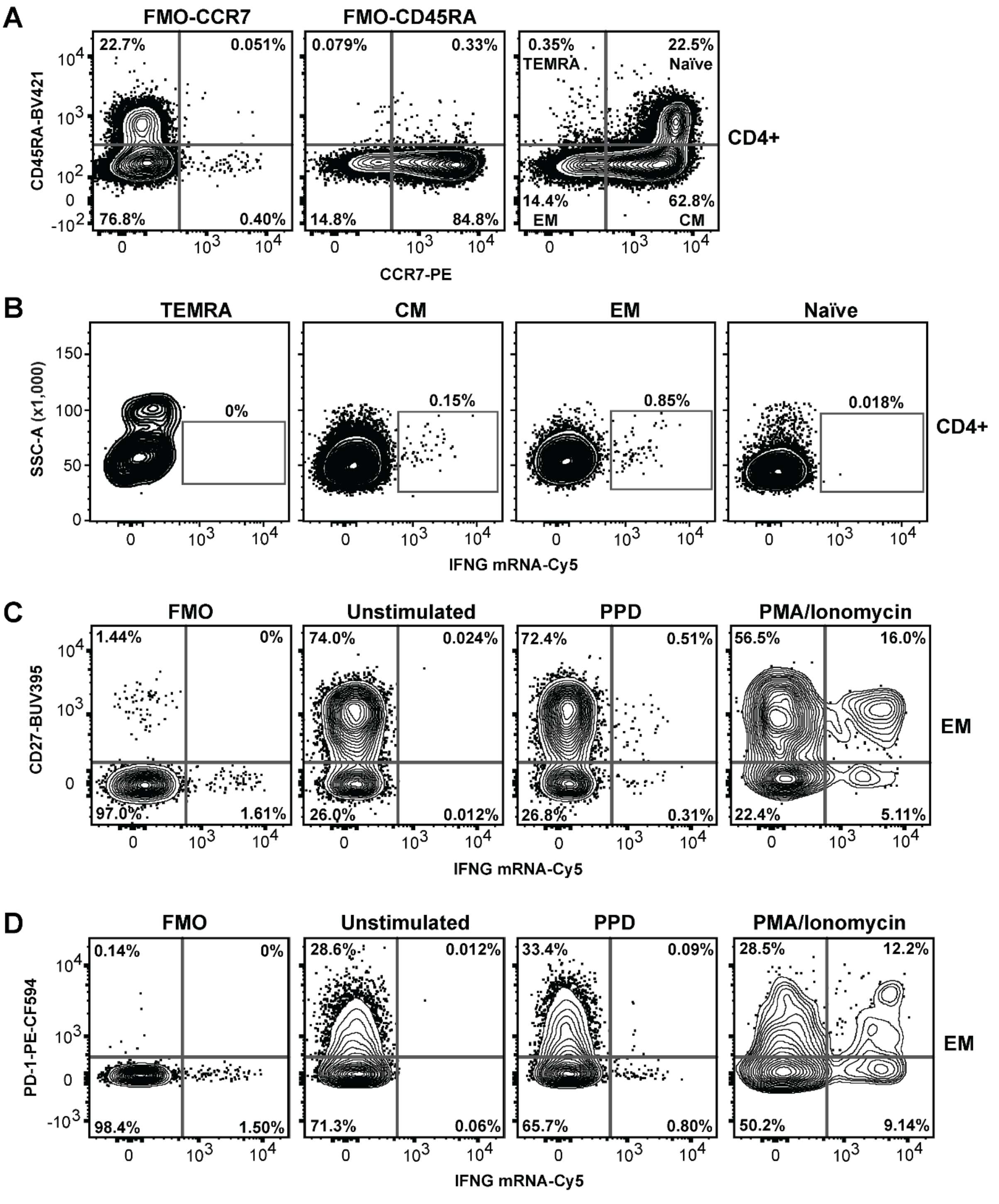

Supplementary Figure 5. Representative gating strategy utilized for the analysis of CD4+ T cell memory subsets. **(A)** Gating strategy utilized for the identification of TEMRA, Naïve, CM and EM subsets in CD4+ T cells. **(B)** IFNG cytokine expression analysis in CD4+ T cells subsets from a representative active TB donor after 6 hrs of PPD stimulation. Gates were defined using unstimulated controls. Cytokine expression in CD27- **(C)** and PD-1+ EM CD4+ T cells **(D)**. The scatter plots show data from one representative active TB donor after 6 hrs of PPD stimulation. Gates were defined according to FMO and unstimulated samples.

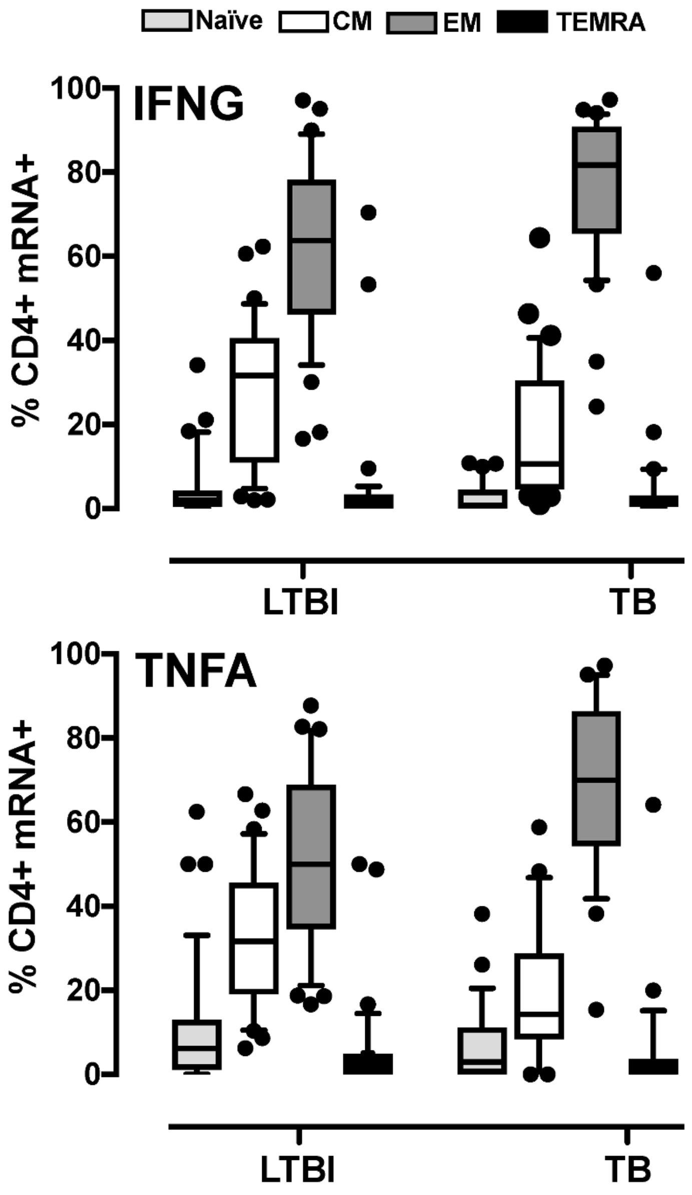

Supplementary Figure 6. Cytokine expression in TEMRA, Naïve, CM and EM CD4+ T cell subsets. Data were expressed as frequency of the total IFNG+ or TNFA+ cells after 6 hrs of PPD stimulation. Similar results were observed at the 2-hr time point (data not shown). The box plots show lower quartile, median, and upper quartile of the distribution. The upper and lower whiskers represent the 90th and 10th percentile. Outliers are shown as (•) symbol.

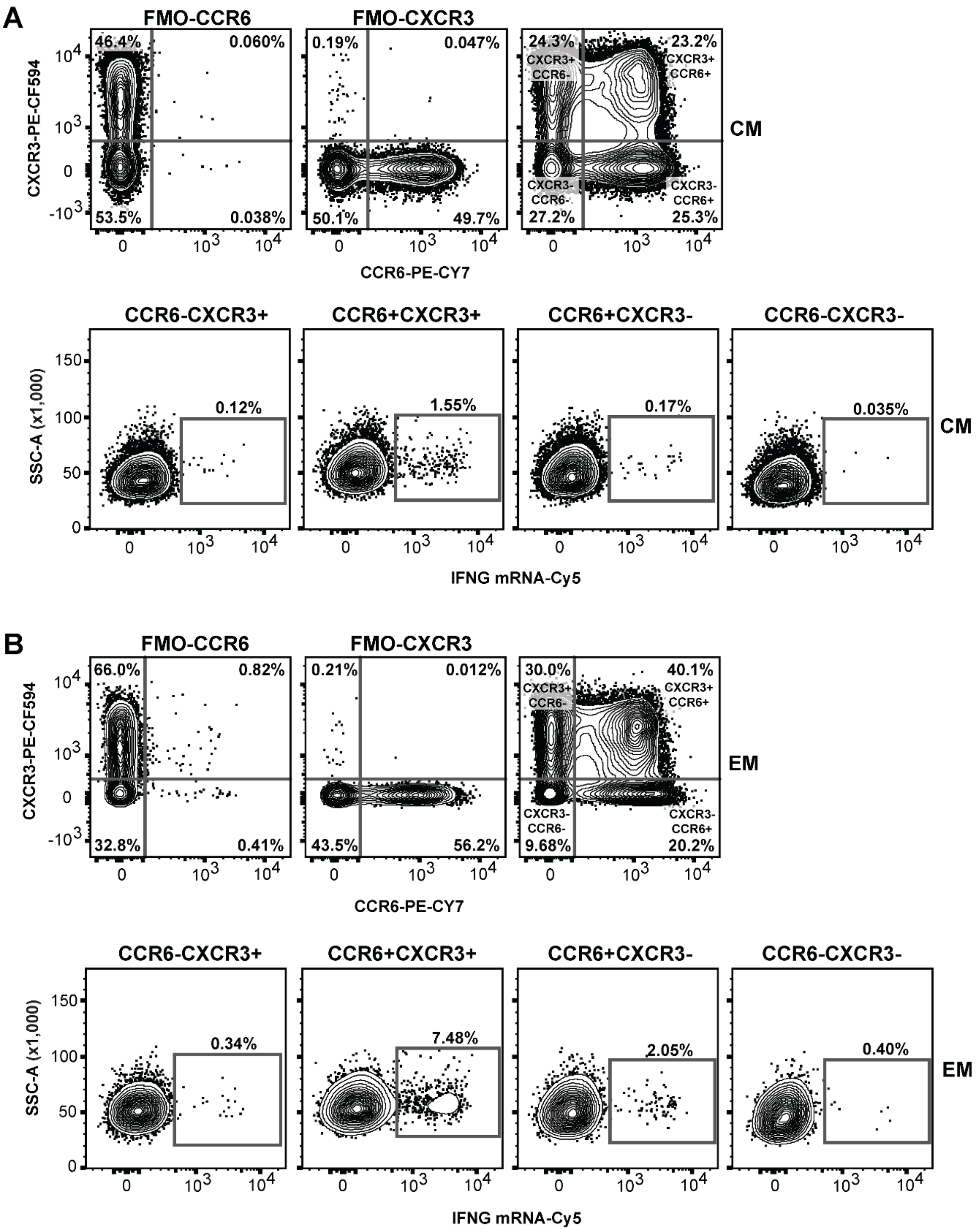

Supplementary Figure 7. Representative gating strategy utilized for the characterization of cytokine expression in CXCR3 and CCR6 memory subsets. IFNG expression in CXCR3 and CCR6 subsets in CM **(A)** and EM **(B)** CD4 T cells. The scatter plots show data from a representative LTBI+ donor after 6 hrs of PPD stimulation. Gates were defined using unstimulated negative controls and FMO controls.

Supplementary TABLE 1. Key demographics of the study population.

**Donor status**		**LTBI+ (n=47)**	**Active TB (n=34)**
**Gender**	Male	25	24
	Female	22	10
**Age (years)**	18-29	5	10
	30-39	12	5
	40-49	12	7
	50-59	9	9
	>60	9	3
**Race**	Asian	9	5
	Black	14	4
	Caucasian	24	25
**Place of Birth**	U.S.A.	12	2
	Central/South America	20	23
	Africa	8	4
	Asia	7	5
	Europe	0	0
